# Evaluation of the Continuous Wavelet Transform for Detection of Single-Point Rub in Aeroderivative Gas Turbines with Accelerometers

**DOI:** 10.3390/s18061931

**Published:** 2018-06-13

**Authors:** Alejandro Silva, Alejandro Zarzo, Juan M. Munoz-Guijosa, Francesco Miniello

**Affiliations:** 1 Department of Applied Mathematics, Escuela Técnica Superior de Ingenieros Industriales, Universidad Politécnica de Madrid, Calle de José Gutiérrez Abascal 2, 28006 Madrid, Spain; a.silvab@alumnos.upm.es; 2 Department of Mechanical Engineering, Escuela Técnica Superior de Ingenieros Industriales, Universidad Politécnica de Madrid, Calle de José Gutiérrez Abascal 2, 28006 Madrid, Spain; juanmanuel.munoz.guijosa@upm.es; 3 Baker Hughes, a GE Company—Bently Nevada, Calle Josefa Valcárcel 26, 28027 Madrid, Spain; Francesco.Miniello@bhge.com

**Keywords:** machine fault diagnosis, rotordynamics, rub, aeroderivative turbines, accelerometers, early fault detection, Fourier analysis, real cepstrum, continuous wavelet transform

## Abstract

A common fault in turbomachinery is rotor–casing rub. Shaft vibration, measured with proximity probes, is the most powerful indicator of rotor–stator rub. However, in machines such as aeroderivative turbines, with increasing industrial relevance in power generation, constructive reasons prevent the use of those sensors, being only acceleration signals at selected casing locations available. This implies several shortcomings in the characterization of the machinery condition, associated with a lower information content about the machine dynamics. In this work, we evaluated the performance of Continuous Wavelet Transform to isolate the accelerometer signal features that characterize rotor–casing rub in an aeroderivative turbine. The evaluation is carried out on a novel rotor model of a rotor–flexible casing system. Due to damped transients and other short-lived features that rub induces in the signals, the Continuous Wavelet Transform proves being more effective than both Fourier and Cepstrum Analysis. This creates the chance for enabling early fault diagnosis of rub before it may cause machine shutdown or damage.

## 1. Introduction

The flawless operation of rotating machinery such as turbomachines plays a key role in many branches of industry such as electrical power and heat generation, aerospace and naval propulsion, and manufacturing. As their performance levels have risen steadily in the course of time their margins of admissible operation have shrunk. A failure in any of these machines could cause considerable economical or even human losses. Thus, it becomes convenient to put in place a maintenance program which allows detecting any abnormality in the machinery operation at the early stages, before a fault at an advance stage forced it to be put out of duty.

**Figure 1:**
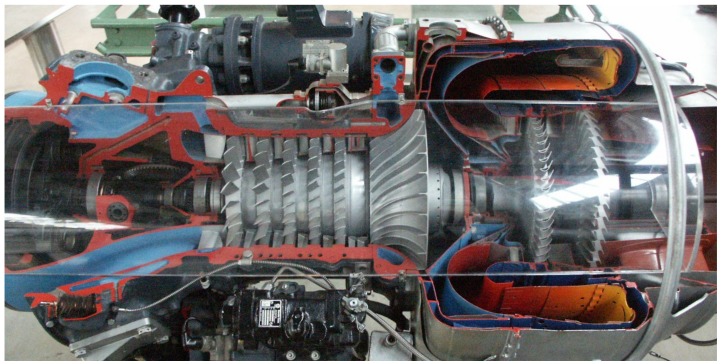
A gas turbine.

In a turbomachine, a *rotor* transmits the mechanical power to or from an external power source or sink. The rotor contains circumferential arrangements of *blades* called *disks* attached to a rotating *shaft*. A *casing* built around the rotor sustains it and channels the working fluid passing through the machine with the aid of disks of *vanes* (Figure 1 or second figure at https://www.power-eng.com/articles/2015/09/ge-announces-launch-of-latest-aeroderivative-gas-turbine-lm6000-pf.html). Optimal thermodynamical efficiency requires, among many other factors, most of the working fluid flow passing through the rotor blades with no leaks. To achieve this, the diametrical clearance between the rotor and the *seals* contained by the casing must be minimized, but the price to pay is a higher probability of rotor–casing rub. This phenomenon causes, when unnoticed, wear and overheating and, if severe, may be highly destructive. Even if modern industrial equipment counts with machine protection relays that shut the machine down in the case of faulty or abnormal duty, a better knowledge of the dynamics of faulty machines—in this case, rotor–casing rub—and more effective monitoring and protection systems lead to earlier diagnosis, shorter downtimes and lower operation and maintenance costs.

**Figure 2:**
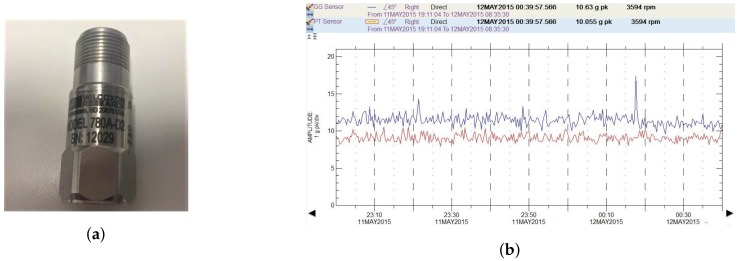
(**a**) A Wilkoxon 780A-D2 general purpose accelerometer for machine vibration monitoring; and (**b**) an accelerometer output representing acceleration as a function of time, in the ADRE monitoring system interface.

Rub dynamics, monitorization, detection and diagnosis of rub and other malfunctions have been research topics in academia for the last decades and industry has large experience on the matter [1,2]. Rotor vibration is the most common indicator of rub available for monitorization. In most gas and steam turbines, vibration is measured with proximity sensors mounted between the rotor and stator. In classes of turbomachinery such as in aeroderivative gas turbines—an elementary part of heat and power co-generation processes, combined-cycle power plants, oil and gas processing and marine propulsion—those sensors cannot be fitted due to several design issues: the very low shaft-bearing relative deformations derived from the use of ball or rolling bearings instead of journal bearings, and the increased difficulty to fulfill the maintenance needs of these sensors. Leveraging on the high casing flexibility of aeroderivative turbines, its operation can instead be monitored by means of indirect vibration measurement techniques such as accelerometers mounted on the casing (Figure 2).

However, despite the industrial relevance of the problem, only a handful of papers have been published on the subject of the detection of rub with acceleration sensors on casing. The authors of [3,4,5] show the results of a series of experimental tests on an experimental aero-engine with a bladed disk and a casing that encloses it. Blades and casing rub with each other when the latter is squeezed onto one of the rotating disk by turning one or several screws. This rub is continuous over some or all of the disc blades in one or several fixed points of the casing. Obviously, if all disk blades rub, the number of impacts per cycle of rotation in each rub point is equal to the number of blades. Casing acceleration is measured at a set of points with accelerometers and spectral analysis is conducted on the data. Raw data and its analysis show periodic rotor–casing impacts taking place with frequency equal to the product of the rotating speed with the number of blades. These impacts have an amplitude-modulated component with frequency equal to rotation speed. Chen [6] contrasted those experimental results with a simulation in a rotor model described in [7]: the shaft is discretized into beam elements; the disk blades are solid, undeformable, straight beams; and the casing is modeled as a two-dimensional curved non-rotating beam with two displacements in the radial plane per node. Wang et al. [8] used another experimental rig to reproduce the same type of rub than in the previous papers: rub is inflicted by means of a nylon block on the casing that impacts with a number of disc blades as the shaft rotates. The data spectra also highlight the impact-frequency peak and the impact modulation, as well as a rotation–frequency amplitude peak and its super-synchronous multiples, clearly seen in the low-frequency spectrum band.

**Figure 3:**
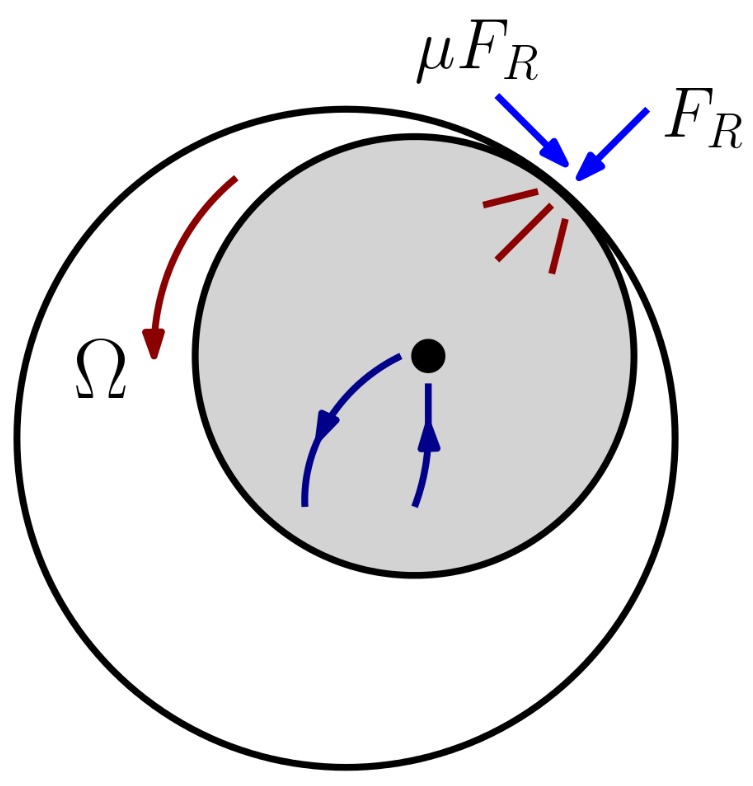
Single-point rotor–casing rub. One rotor spot impacts one point on the casing at a time. During the contact, normal and friction rub forces (light blue) change the rotor orbit direction (dark blue) and inflict vibration in the system.

The publications cited in the previous paragraph are centered on the same type of rub: with the infliction of a massive relative rotor–casing mechanical deformation at one or several fixed points, or by fitting an obstacle between rotor and casing, the authors reproduce a rub that extends through all or almost all the circumferential rotor disk blades. There is an interest in the study of lighter and more frequent types of rub such as partial, single-point rub (See chapter 2 of [2]): one rotor spot impacting a single point at the casing (Figure 3). At the same time, there is an opportunity to study the dynamics of longer, flexible casings in medium or heavy-duty turbomachinery and the influence of the axial positions of casing sensors and rub points on the collected signals. The latter could be attained with the construction of larger experimental rotor–casing rigs and their modelization as three-dimensional shells instead of using curved beams in the radial plane.

This research is centered on aeroderivative gas turbines due to their increasing importance and the industrial demand for effective rub detection in these machines. The rotating speeds of aeroderivative turbines during steady-state regime use to lie between the first and second rotor critical speeds, far lower than the regimes of most aero-engines. In most cases reported by the industry, rotor–casing rub leading to rotor shutdown took over in that regime, thus, in this work, the focus was on the identification of possible rub in the duty range of aeroderivative turbines. Any rub regime in a rotating machine is, at the onset, a light rub whose severity may increase in time if no measure is taken, therefore the best signal processing tools used in the task of diagnosing rub should be well suited to the lightest rub that can be measured and detected. Knowledge of the consequences of the lightest types of rotor–casing rub on machine dynamics and vibration is necessary to be able to detect it and take corrective action as soon as possible. With early diagnosis, further damage can be prevented and maintenance programs and operation costs can be optimized.

In this work, the performances of the Fourier Transform and the Cepstrum [9], the standard tools used by the Industry for the monitorization and diagnosis of malfunctions in machinery, are compared to the performance of the Continuous Wavelet Transform [10] for the detection of single-point rub in aeroderivative turbines. Wavelet Analysis is still unfamiliar to most industrial practitioners, but it has been extensively used in the scientific state of the art for machine fault diagnosis [11] and therefore is a promising alternative to the traditional machine diagnosis methods. To do so, a novel rotor model with flexible casing and supports, presented in Section 2, was developed. With this model, we carried out the simulation of a rotating machine subject to rotor unbalance and rotor–casing rub. This model is able to reproduce many types, conditions and severities of rub but the results described here revolve around single-point rub—the most common type of rub diagnosed in turbomachinery (see Figure 3). The results of those simulations are described in Section 3: the model equations were integrated and vectors of casing acceleration and velocity samples—or *time records*—were extracted and processed with the methods cited above. The performance of the three signal processing methods can be measured by the degree of rub features that each method can isolate, the computational resources they consume, and by the time necessary to identify rub features on the signals since the onset of a rub condition in the machine. In the presence of rub the time-domain acceleration signals consist of trains of impulses followed by oscillating transients that contain damped rotor and casing vibration. All those features in both low- and high-frequency bands may be visible to the Fourier spectra and the cepstra only if the sampling times are large enough, but the Continuous Wavelet Transform can highlight and localize these features in a short sampling vector containing just a few rotor–casing impacts, confirming the existence of a periodical impact on the casing. This work goes in the direction to find tools that allow for rub diagnosis while its severity it still very small to prevent machine damage or fault—i.e., early fault diagnosis.

## 2. Materials and Methods

The models, simulations and algorithms described here have been implemented in the Matlab environment, Release 2017a, under a 64-bit system with 8 GB of RAM, an x64 2.4 GHz Intel Core processor and Windows 10 Enterprise as operating system.

### 2.1. Rotor–Casing Model

**Figure 4:**
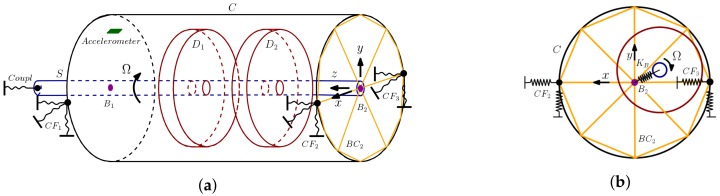
The rotor–flexible casing model: (**a**) perspective view; and (**b**) radial view of the model end, with the rotor displaced from the bearing node $B_{2}$. $K_{B}$ represents bearing stiffness.

The rotor–flexible casing model that has been developed (Figure 4) comprises a cylindrical shaft (*S*, blue color) with a number of cylindrical rigid solid disks ($D_{1}$ and $D_{2}$, red color) attached to it. Together, they form the machine rotor, which rotates at a constant speed (Ω). The rotor is supported on bearings ($B_{1}$ and $B_{2}$, violet) that are radially bound to the cylindrical shell that forms the machine casing (*C*, black). In an aeroderivative gas turbine, the ends of a casing are attached to solid elements called *frames*, which also house the rotor bearings. These bearing-casing joints are modeled as very stiff bar trusses between each bearing and a number of homogeneously-distributed points in the casing ($BC_{1}$, not represented for simplicity; and $BC_{2}$, yellow). All components are modeled under the hypotheses of linear elasticity (with the exception of the rigid solid disks), homogeneity and small displacements and deformations. Any of the two shaft extremes may incorporate a linear elastic coupling attached to the ground at the other side (*coupl*, dark blue). The casing rests on the ground through linearly elastic supports attached to several points of the casing ($CF_{4}$, not represented; and $CF_{1}$, $CF_{2}$ and $CF_{3}$, black). The *z* axis of the global cartesian reference system (black) follows the rotor axial direction and the *x* and *y* axes are contained in a rotor radial plane at one of the shaft extremes. A positive rotor rotation speed follows the shortest path from *x* to *y*.

This rotor may vibrate due to the action of unbalance and rub forces that will be described further below. The motions caused by those forces are measured by one or several accelerometers on the casing (green) which yield casing acceleration versus time. The rotor radial motion—called the rotor *orbit*—is also of great interest because that vibration is what is monitored in conventional turbomachines as the indicator of malfunction.

This model can be adapted to any material properties, dimensions and numbers of disks, bearings and supports, as long as the model hypotheses hold. As shown in Figure 4, our model was set up with two disks, two rotor bearing supports at both casing sides, a coupling at one of the shaft extremes and four casing-ground supports attached to the casing ends in a plane that splits the casing in two identical halves. Shaft and disks are made of steel. The casing’s mechanical properties correspond to carbon fiber. Bearing–casing joints are modeled as very rigid elements so that most of the bearing-casing union elasticity rests on the bearings themselves. The most remarkable dimensions and mechanical properties of the model are shown in Table 1. The values in Table 1 are within the range of those appearing in some reduced scale experimental devices typically employed in the literature for model validation [6,12].

The model simulation requires the dynamical system of ordinary differential equations (SODE) shown in Equation (1). In this work, it was derived with the Finite-Element Method following the model hypotheses. A thin, slender shaft has been discretized into two-node Euler–Bernuolli beam elements with five degrees of freedom per node: three translations and two bending rotations. Mass, gyroscopic and stiffness local matrices of a two-node Euler beam element were taken from [13], with the neglect of the torsional degrees of freedom. The rigid solid disks—modeled as in [14]—share these five degrees of freedom with the shaft. The casing was split into a grid of equally-sized Mindlin–Reissner shell elements with four nodes at the element corners and two additional nodes at the radial boundaries to allow the isoparametric modeling of a curved cylindrical shell. The formulation we followed to derive the local equations of casing elements is in [15] and is based on treating each shell element as a degenerate 3D element under shell hypotheses, with nodes only in the midplane. Each casing node has five degrees of freedom: the three cartesian translations and two bending rotations around the in-plane local axes, as usual in shell elements. The bearing nodes and those of the stiff bars that bind them to the casing only have as degrees of freedom the three translations in space. As bearing hypotheses, there are no cross-coupled reaction forces between bearings and shaft and the shaft is supposed to rotate freely in the bearing, with no reaction forces caused by relative bearing-shaft angular deformations. This hypotheses are applicable when the shaft is supported on ball or roller bearings, as it is in most aeroderivative turbines.

(1) $$\begin{array}{c} Mu^{\prime \prime }+\left(C+\Omega G\right)u^{\prime }+Ku=F_{U}\left(t\right)+F_{R}\left(u,u^{\prime }\right) \end{array}$$

**Figure 5:**
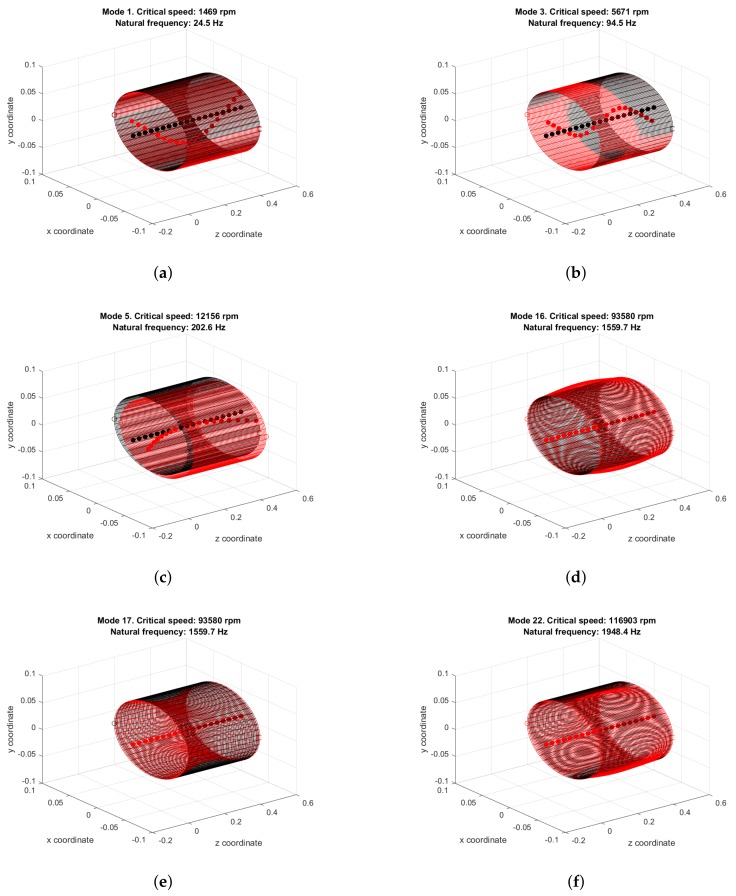
Lowest shaft and casing natural modes: (**a**) rotor bending mode with one antinode; (**b**) rotor bending mode with two antinodes; (**c**) casing rigid solid mode with shaft deflection; (**d**) lowest casing longitudinal mode; and (**e**,**f**) lowest casing flexural modes.

M, C, G and K are the mass, viscous damping, gyroscopic and stiffness matrices, respectively. These global matrices are assemblies of the local matrices of shaft and disk (*S+D*), casing (*C*) and bearings (*B*). The viscoelastic couplings between shaft and bearings and between bearings and casing are accounted for with the addition of elements outside the main diagonal to the global viscous damping and stiffness matrices:

$$\begin{array}{c} \begin{array}{cc} M=\left[\begin{array}{ccc} M_{S+D} & 0 & 0\\ 0 & M_{C} & 0\\ 0 & 0 & M_{B} \end{array}\right] & G=\left[\begin{array}{ccc} G_{S+D} & 0 & 0\\ 0 & 0 & 0\\ 0 & 0 & 0 \end{array}\right]\\ C=\left[\begin{array}{ccc} C_{S+D} & 0 & C_{S+D,B}\\ 0 & C_{C} & C_{C,B}\\ C_{B,S+D} & C_{B,C} & C_{B} \end{array}\right] & K=\left[\begin{array}{ccc} K_{S+D} & 0 & K_{S+D,B}\\ 0 & K_{C} & K_{C,B}\\ K_{B,S+D} & K_{B,C} & K_{B} \end{array}\right]. \end{array} \end{array}$$

u, $u^{\prime }$ and $u^{\prime \prime }$ are the vector of node displacements and its time derivatives: velocity and acceleration. Analogously to the matrices, these vectors are assemblies of local vectors of shaft, casing and bearing displacements, each with its own degrees of freedom:

$$\begin{array}{c} u=(u_{S+D}|u_{C}{\left|u_{B}\right)}^{T}={\left(u_{1}\;u_{2}\;\ldots \;u_{N}\right)}^{T}. \end{array}$$

where *N* is the total number of nodes. Simulations were carried out with a mesh of 19 shaft nodes, 18 shaft beam elements, 2 bearing nodes, 5490 casing nodes, and 2700 casing shell elements. The first step was a modal analysis of the free undamped system with Ω=0. The first rotor and casing modes of vibration and their corresponding undamped natural frequencies are shown in Figure 5.

Following [7], the system has been hypothesized to have a Rayleigh damping: the global viscous damping matrix C as a linear combination of the mass and stiffness matrix:

$$\begin{array}{c} C=C_{M}M+C_{K}K. \end{array}$$

The two constants $C_{M}$ and $C_{K}$ are the result of solving a two-variable system of equations given two system natural frequencies $\omega_{N,1}$ and $\omega_{N,2}$ with known damping factors $\xi_{d,1}$ and $\xi_{d,2}$:

$$\begin{array}{c} \xi_{d,i}=\frac{1}{2}\left(\frac{C_{M}}{\omega_{N,i}}+C_{K}\omega_{N,i}\right),\;i=1,2. \end{array}$$

**Figure 6:**
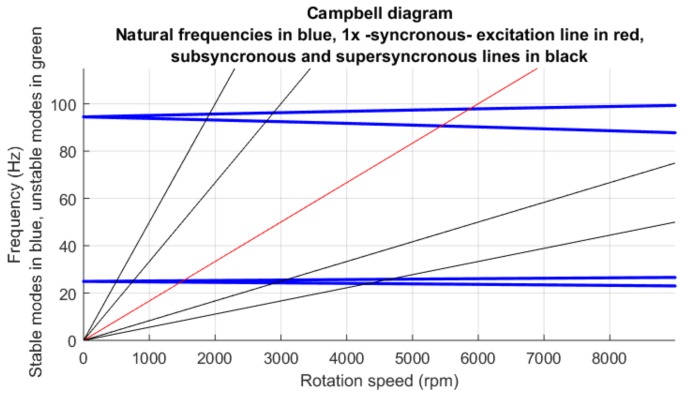
Campbell diagram of the two first rotor natural modes. The two bending modes split with increasing rotation speed due to the gyroscopic effect. The intersections of the 1× line with the natural modes mark the *resonance* or *critical* speeds of the rotor. All represented modes are stable.

The variation of natural frequencies with rotation speed in the damped free rotor–flexible casing due to the speed-variable damping term of Equation (1) is represented on the *Campbell diagram* shown in Figure 6.

#### 2.1.1. Rotor Unbalance

The most common source of excitation in a rotating machine is an unbalance force in the rotor (See chapter 18 of [2]). The unbalance force vector at shaft node *i* is:

$$F_{U},i=M_{U,i}\Omega^{2}r_{U,i}{\left(\cos\left(\Omega t-\psi_{U,i}\right)\;\sin\left(\Omega t-\psi_{U,i}\right)\;0\;0\;0\right)}^{T},$$

where $M_{U,i}$ is an unbalance mass, $r_{U,i}$ is the distance between the unbalance mass and the rotation axis and $\psi_{U,i}$ is a phase angle that defines the angular position of the unbalance mass with respect to a reference point at the rotor. In rotating machines, this reference may be the *keyphasor* mark (See chapter 2 of [2]).

#### 2.1.2. Rub Forces

**Figure 7:**
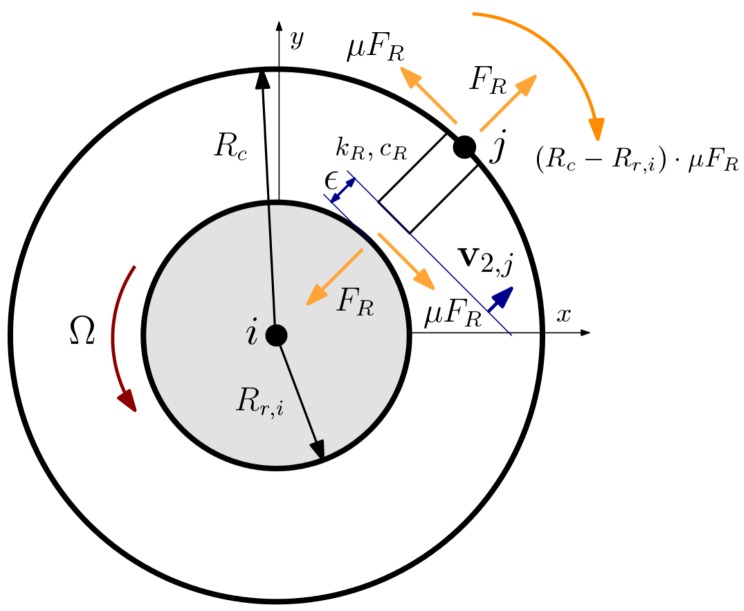
Rub model and forces at shaft and casing nodes.

A number of rotor–casing rubs can be defined, each between a casing node and a rotor node (Figure 7). An obstacle with stiffness and damping coefficients $k_{R}$ and $c_{R}$ is fitted at the casing node *j*. This obstacle may impact the rotor at node *i*. When the system is at rest, the clearance between rotor and obstacle is ϵ. If the rotor–casing relative deformation becomes larger than ϵ, two forces appear in the system: a normal, viscoelastic force ($F_{R}$) and a tangential friction force proportional to the normal rub force by a friction coefficient (μ). This friction coefficient can be considered constant if the contact surfaces never stick with each other and if changes in the relative rub velocity during contact are small.

Fixed-point rub models such as this one have been studied in some publications: Ma et al. [16] and Behzad et al. [17] applied Lagrangian methods to model the point-to-point contact between an undeformable rotor surface and an elastic rod that disturbs the rotor motion. The model formulations define a gap function between the two surfaces with a master–slave approach and enforce a no-penetration condition. The models were simulated at subcritical (below the first rotor critical speed) and supercritical (above the first rotor critical speed) rotation speeds. Following the same approach, Ma et al. [18] then used the same formulation to study fixed-point rub with three-pin–three rub spots and four-pin–four rub spots stators. Mokhtar et al. [19] added rotor torsional deformations to a model similar to those of the aforementioned references, concluding that the spectra of torsional vibration, with the presence of multiples of the rotation speed and the excitation of the torsional natural frequencies, may be indicative of rub.

If the obstacle contact surface is flat and parallel to the casing at the attachment node *j*, its normal unitary vector equals the casing unitary normal vector at *j*, $v_{2,j}$.

The relative displacement $d_{R}$ between rotor and obstacle and the relative velocity $d_{R}^{\prime }$ can be expressed as:

$$\begin{array}{c} \begin{array}{c} d_{R}=\langle \left(u_{x,j}-u_{x,i}\;u_{y,j}-u_{y,i}\right),\;v_{2,j}\rangle -\epsilon \\ d_{R}^{\prime }=\langle \left(u_{x,j}^{\prime }-u_{x,i}^{\prime }\;u_{y,j}^{\prime }-u_{y,i}^{\prime }\right),\;v_{2,j}\rangle , \end{array} \end{array}$$

being $u_{x,j}$, $u_{y,j}$, $u_{y,i}$ and $u_{x,i}$ the radial displacements of the casing and rotor nodes respectively. $u_{x,j}^{\prime }$, $u_{x,i}^{\prime }$, $u_{y,j}^{\prime }$ and $u_{y,i}^{\prime }$ are node velocities. 〈.,.〉 is the notation for the inner product of two vectors.

The normal rub force results from the product of relative displacements and velocities with stiffness and damping contact coefficients $k_{R}$ and $c_{R}$ and a Heaviside function that equals 1 when the relative deformation is larger than the clearance ϵ, and 0 otherwise:

$$\begin{array}{c} F_{R}\left(u,\;u^{\prime }\right)=\left(k_{R}d_{R}+c_{R}d_{R}^{\prime }\right)\cdot H\left(d_{R}-\epsilon \right). \end{array}$$

Since the rub spot stands at a distance $R_{c}-R_{r,i}$ of the casing node, $R_{r,i}$ being the rotor radius at shaft node *i*, the friction force at the rub spot inflicts, by balance of forces, a bending moment at the casing around the *z* axis. Normal and friction rub forces at rotor and casing are projected over the global coordinate axes, resulting in the following local rub vectors of rotor (node *i*) and casing (node *j*):

$$\begin{array}{c} \begin{array}{c} F_{R},i\left(u,u^{\prime }\right)=F_{R}\left(\begin{array}{c} -\cos\gamma \\ -\sin\gamma \\ 0\\ 0\\ 0 \end{array}\right)+\mu \cdot F_{R}\left(\begin{array}{c} \sin\gamma \\ -\cos\gamma \\ 0\\ 0\\ 0 \end{array}\right)\\ F_{R},j\left(u,u^{\prime }\right)=F_{R}\left(\begin{array}{c} \cos\gamma \\ \sin\gamma \\ 0\\ 0\\ 0 \end{array}\right)+\mu \cdot F_{R}\left(\begin{array}{c} -\sin\gamma \\ \cos\gamma \\ 0\\ -(R_{c}-R_{r,i})\\ 0 \end{array}\right), \end{array} \end{array}$$

where γ is the angle of $v_{2,j}$ with respect to *x*.

### 2.2. Model Reduction and Integration

#### 2.2.1. The Craig–Bampton Method

The model mesh counts with a total of 27,551 degrees of freedom, making a numerical integration of model Equation (1) too complicated for the limited speed and storage available and the large size of the integration output. Before its numerical integration, the system should be reduced in size. This can be attained with the Craig–Bampton method [20].

A description of the Craig–Bampton method as applied to our problem can be found in Appendix A.

As boundary degrees of freedom, we chose those of the shaft, the casing degrees of freedom affected by rub and degrees of freedom selected as data source for the measurement of casing acceleration. The interior degrees of freedom were projected over a subspace whose basis is formed by the first 200 normal modes of a system with constrained boundary degrees of freedom. The resulting reduced system has only 300 degrees of freedom, a size a modern desktop computer can cope with in terms of storage and speed.

#### 2.2.2. The Newmark-β Methbd

For the numerical integration of the reduced system of equations, we chose the implicit Newmark-β method [21]. This method uses, in an iterative fashion, a implicit finite-difference expression to calculate the velocities and displacements of time step i+1 from the displacements, velocities and accelerations of the former step *i* and the accelerations of the current step i+1. The Newmark-β method is for application to linear dynamical systems, but Equation (1) contains a strongly nonlinear force term $F_{R}$. This problem can be circumvented with the use of nonlinear algebraic solvers to clear the i+1-th step velocities and displacements contained in the force term but a much simpler alternative is to substitute the state vector $\left(u,u^{\prime }\right)$ of the rub force term for the displacements and velocities calculated in the previous *i*-th step:

$$\begin{array}{c} F_{R,\mathrm{CB}}\left(u_{i+1},u_{i+1}^{\prime }\right)\approx F_{R,\mathrm{CB}}\left(u_{i},u_{i}^{\prime }\right). \end{array}$$

### 2.3. Signal Extraction and Processing

#### 2.3.1. Fourier Analysis

A vector x of periodical discrete signal samples with length *N* can be decomposed into a Fourier sum series with sinusoidal terms called *harmonics*. The *N* coefficients X(n) of the decomposition form the signal *spectrum* and are calculated with the *Discrete Fourier Transform* (DFT) (see Vetterli, M.; Kovačević, J. Goyal, V. K. [22]). The sampling vector x contains, in this work, the radial acceleration of a casing node measured with respect to time, being $T_{s}$ its sampling period. Before the spectrum calculation, the signal is periodizised, multiplying it element-wise with a Hamming window vector. If *N* is a power of two, the *Fast Fourier Algorithm* (FFT) (see Cooley, J. W. Tukey, J. W. [23]) is applicable, allowing for a much faster computation of the spectrum.

#### 2.3.2. Real Cepstrum

The *cepstrum* [9], also known as the *spectrum of the spectrum*, is a popular diagnosis tool in machinery condition monitoring. Generally, it is the inverse Fourier transform of the logarithm of a spectrum. In the case of the *Real Cepstrum*, its mathematical definition is:

$$\begin{array}{c} c\left(t\right)=F^{-1}\left(\log\left|F(x(t)\right)|\right). \end{array}$$

First, the complex phase information of the signal x(t) is removed with the calculation of the module of its spectrum, |F(x(t))|. Then, the logarithm evens out the amplitude of any sidebands or impulse trains contained in the spectrum with the amplitudes of the main spectral components. After the application of the inverse Fourier transform, impulse trains and amplitude or frequency-modulated components contained in the original signal are highlighted in the resulting cepstrum and shown as periodical pulses called *rahmonics* with a time-dimensional periodicity called *quefrency*, measured in seconds.

#### 2.3.3. Continuous Wavelet Transform

Analogously to the Continuous Fourier Transform (see Kovacevic, J.; Goyal, V. K. Vetterli, M. [10]) the *Continuous Wavelet Transform* (CWT) is the inner product of a function x(t) and a dilated and translated basis function Ψ called *mother wavelet*:

$$\begin{array}{c} W\left(a,b\right)=\langle x\left(t\right),\;\Psi_{a,b}\left(t\right)\rangle =\frac{1}{\sqrt{\left|a\right|}}\int_{-\infty }^{\infty }x\left(t\right)\cdot \Psi^{*}\left(\frac{t-b}{a}\right)\;dt, \end{array}$$

where *a* and *b* are, respectively, dilation and translation factors. The coefficient W(a,b) gives a measure of the similarity between the function and the dilated and translated mother wavelet. The big advantage of the CWT over the Fourier Analysis is its potential for feature localization in both time and frequency domains, provided that the selected mother wavelet has good properties—smoothness, compact support, regularity, etc. Hence, it is ideal for signals with a high content in transient components and irregularities that are invisible to the Fourier decomposition methods. CWT analysis with the Morlet function as the mother wavelet [24] has been successfully used as a tool for machine fault diagnosis in former studies [25,26,27,28,29,30,31].

To improve the resolution of the wavelet scalograms, we applied a *synchrosqueezing* frequency reassignment to the wavelet coefficients. This post-processing algorithm, described by Daubechies et al. in [32], corrects the smearing of energy in the wavelet decomposition by concentrating the spectral energy closer to the instantaneous frequency at each instant of time. The result is a less blurred representation of the frequency content of the signals across time.

## 3. Results

The simulated rotor–casing model comprises two disks symmetrically attached to the shaft. The second disk contains an unbalance mass $M_{U}$ of $1.5\;\times 10^{-3}$ kg at a distance $r_{U}$ of $3.75\times 10^{-2}$ m from the rotation axis with zero phase angle $\psi_{U}$. The viscous damping matrix has been estimated considering a damping factor of 0.04 and 0.06 at the critical speeds of 1500 and 5700 rpm, respectively. A rub condition between the rotor and an element attached to the casing affects a shaft node close to one of the disks and one of the casing nodes in the same radial plane, with $k_{R}=2.25\times 10^{6}$ N m${}^{-1}$ being the average stiffness of a seal strip tested as rub obstacle in [33], $c_{R}=0$ being no impact damping and μ=0.2 being steel-to-steel rub. Equation (1) is integrated with a time step of $2^{-17}$ s. Such small time step is necessary to guarantee the stability of the numerical solutions, otherwise spurious oscillations would blur the high-frequency casing vibration harmonics. Casing radial acceleration of a casing node close to one of the casing ends is extracted into a vector of $2^{18}$ samples at a rate of one sample per integration step, the sampling time of the collected acceleration data or *time record* being 2 s. The radial acceleration of casing node *j*, $u_{j}^{\prime \prime }$, as a casing accelerometer measures it is defined by its acceleration in the *x* and *y* degrees of freedom as $\sqrt{u_{x,j}^{\prime \prime }+u_{y,j}^{\prime \prime }}$.

It is common practice to integrate the accelerometer signals, monitoring velocity instead of acceleration with respect to time. The purpose is to attenuate the high-frequency noise contained in the accelerometer signal and to highlight the low-frequency band of the spectrum, which is the source of information that is most commonly used for protection purposes in rotating machinery [34]. Therefore, a second sampling vector containing radial casing velocity, $u_{j}^{\prime }$, is constructed from the simulation data. Rotor displacement orbits in the radial plane where it rubs the casing are also of interest because in conventional turbomachines rotor orbits are the preferred monitorization tool. Rotor orbits bring us confirmation of the existence of a rub in the simulated rotor–flexible casing model.

From the Campbell diagram (Figure 6), the first three rotor critical speeds are approximately 1500 rpm, corresponding to the rotor bending mode with one antinode, and 5400 and 5900 rpm corresponding to the bending mode with two antinodes. The model was simulated at a speed Ω of 3600 rpm (60 Hz), between the first and the second critical speeds: the working range of a real aeroderivative machine. The purpose of selecting this rotation speed is to reproduce a rub condition that is observed very often in conventional turbomachines working at speeds above twice the first rotor critical speed: a single-point rub with one impact every two rotation cycles (See chapter 2 of [2]). This period-two regime under supercritical rotation speeds is also observed in other works that deal with single-point—or fixed-point—rub [16,17,19].

**Figure 8:**
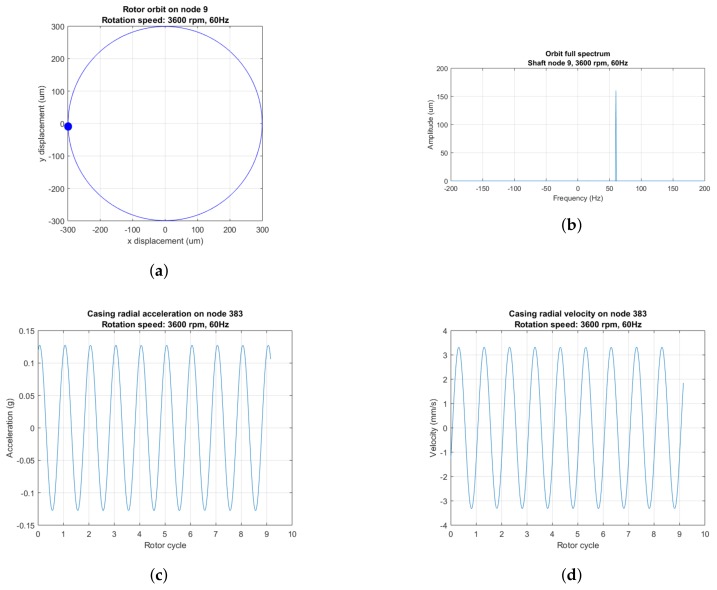
(**a**) Rotor orbit shaft displacement in the radial plane; (**b**) full spectrum of the rotor orbit; and (**c**,**d**) acceleration versus rotor cycle, i.e., versus time, and velocity versus rotor cycle—“acceleration time plot” and “velocity time plot”, respectively, hereafter—measured at selected casing node at 3600 rpm, with no rub and the shaft rotating in the anticlockwise direction.

**Figure 9:**
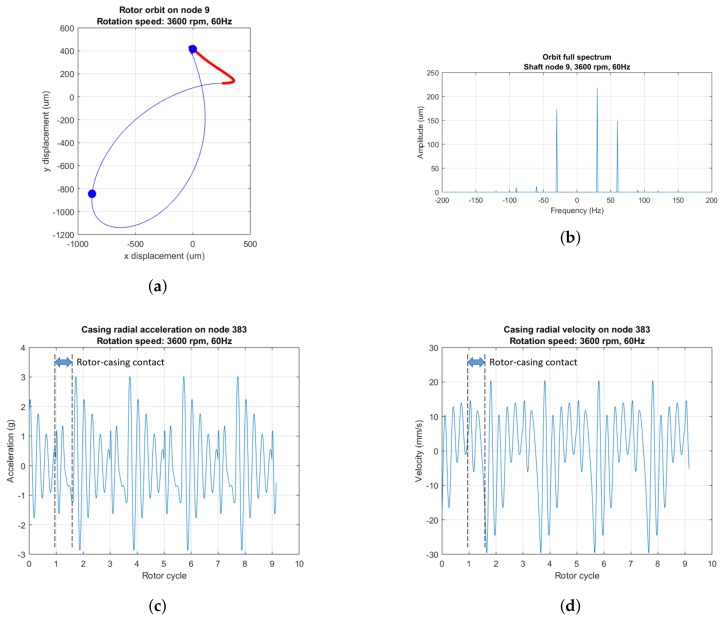
(**a**) Rotor orbit, with the orbit segment where the rotor rubs the casing marked in red; (**b**) full spectrum of the rotor orbit; (**c**) acceleration time plot; and (**d**) velocity time plot at 3600 rpm with single-point rub ($\epsilon =2.5\;\times \;10^{-4}$ m). The shaft rotates in the anticlockwise direction. In (**c**,**d**), black dotted lines mark the boundaries of the leftmost rotor–casing contact.

In Figure 8 and Figure 9, we represent the rotor orbits, the full spectra of the rotor orbits (See chapter 8 of [2]) and casing acceleration and velocity plots of a rotor–casing model in steady-state regime under two conditions: no rub and single-point rub. If there is no rub (Figure 8), the acceleration and velocity signals are dominated by a synchronous sinusoid resulting from the unbalance force excitation. If the clearance ϵ is small enough, a stable single-point rotor–casing sets off, its intensity being of such magnitude so that the unbalance-related sinusoidal vibration is masked by new signal features resulting from the rub condition.

Every time the rotor impacts the casing, two impulse peaks appear in the acceleration plot (Figure 9c): the first when rotor and casing come together, and the second when rotor and casing come apart. A transient that contains at most free high-frequency highly-damped vibration of rotor and casing follows the first impulse. The second impulse at the instant when rotor and casing split apart is followed by a damped transitory dominated by the 200 Hz casing rigid solid mode (Figure 5c). The existence of these two sharp impulses at the boundaries of rub contact and the damped high-frequency oscillations following can be confirmed by results found in the literature as the stator lateral response plots of [19]. The number of rotor–casing impacts per second is half the rotation frequency: 30 Hz.

A single-point rub regime is easy to identify in the full spectrum plots (Figure 9b): it manifests itself as a pair of harmonics—forward and backward—with frequency half the rotation speed and equal or almost equal amplitude. This harmonic with half the rotation speed is a staple in the spectra of rubbing rotating machinery operating at speeds slightly above twice the first critical speed (See chapter 2 of [2]). The construction of full spectra of rotor orbits requires the relative displacements between rotor and stator in at least one rotor radial plane. Unfortunately, that information cannot be collected in an aeroderivative turbine because of the technical challenge that means to fit proximity sensors in aeroderivative gas turbines.

In the following sections, the signal processing methods described above are applied to the casing signals from the rotor with light rub (Figure 9) to isolate the signal features that allow for rub diagnosis.

### 3.1. Fourier Analysis

**Figure 10:**
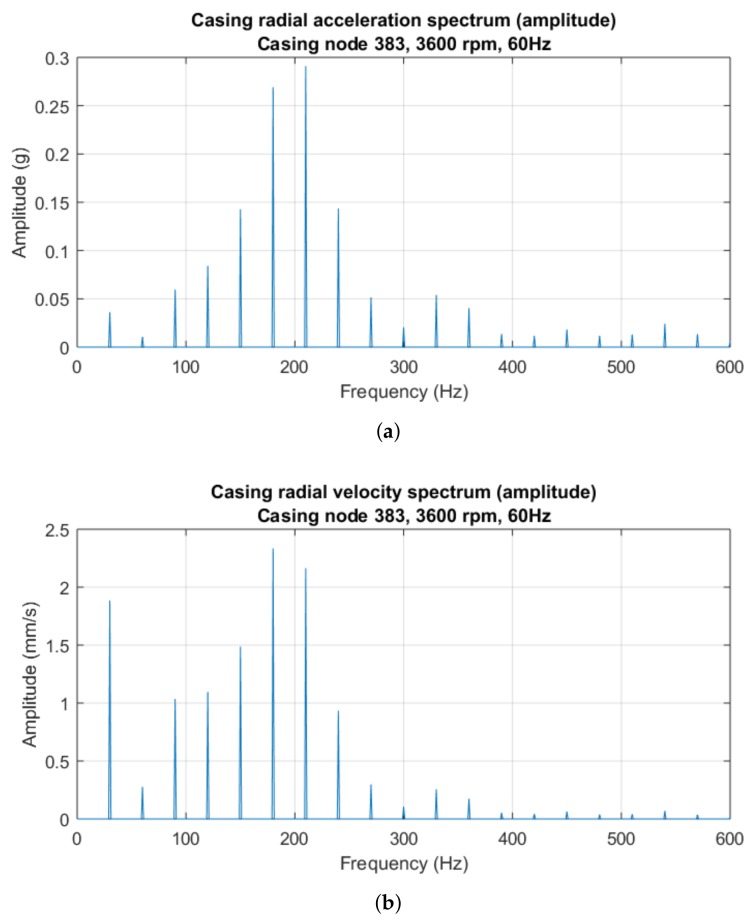
Periodograms of the steady-state casing signals with single-point rub and low-band details: (**a**) casing acceleration; and (**b**) casing velocity. For simplicity, only the low-band side of the spectra—between 0 and 600 Hz—is shown.

The Fourier spectra of the acceleration and velocity records under single-point rub are shown in Figure 10. Once a low-pass FIR filter was applied to the discrete sampling vectors to prevent aliasing, the acceleration data were downsampled to 28 kHz to yield a 0–14 kHz spectrum, and the velocity data were downsampled to 2 kHz for a 0–1000 Hz spectrum. Then, a Hamming windowing was applied to the new vectors of samples. The two sample records were two seconds long, hence the frequency resolution is 0.5 Hz. The two spectra are qualitatively very similar: each displays an impulse train with fundamental frequency equal to the rotor–casing impact frequency—half the rotation speed, 30 Hz. The harmonics with the highest amplitude, apart from the fundamental harmonic in the velocity spectrum, are found in the 90–240 Hz band and are related to the excitation of the 200 Hz casing rigid solid vibration mode (Figure 5c). This amplification phenomenon of the rub frequency harmonics near the casing natural frequencies has already been observed in publications (e.g., [35]).

### 3.2. Real Cepstrum

**Figure 11:**
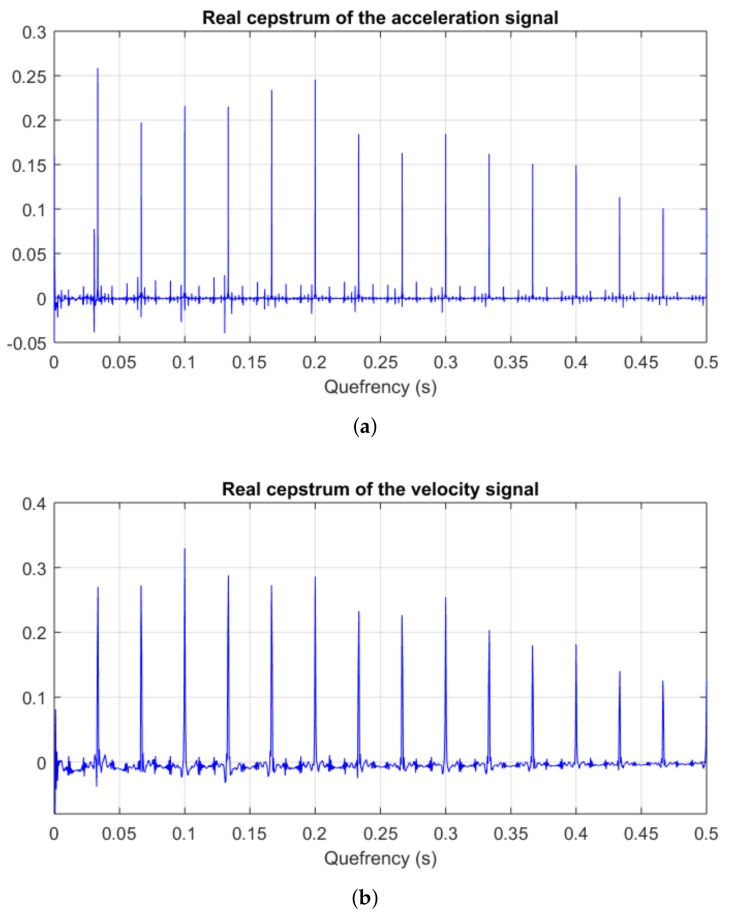
Real cepstra of the accelerometer signals with single-point rub: (**a**) acceleration signal; and (**b**) velocity signal.

The cepstrum analysis of the downsampled and filtered acceleration and velocity signals (Figure 11) underlines the rotor–casing impact frequency of 15 Hz—represented in the cepstra as impulse trains of quefrency 0.033 s. Both cepstra are qualitatively and quantitatively very similar, although the velocity cepstrum looks coarser due to the lower sampling frequency of the downsampled velocity data.

### 3.3. Continuous Wavelet Transform

**Figure 12:**
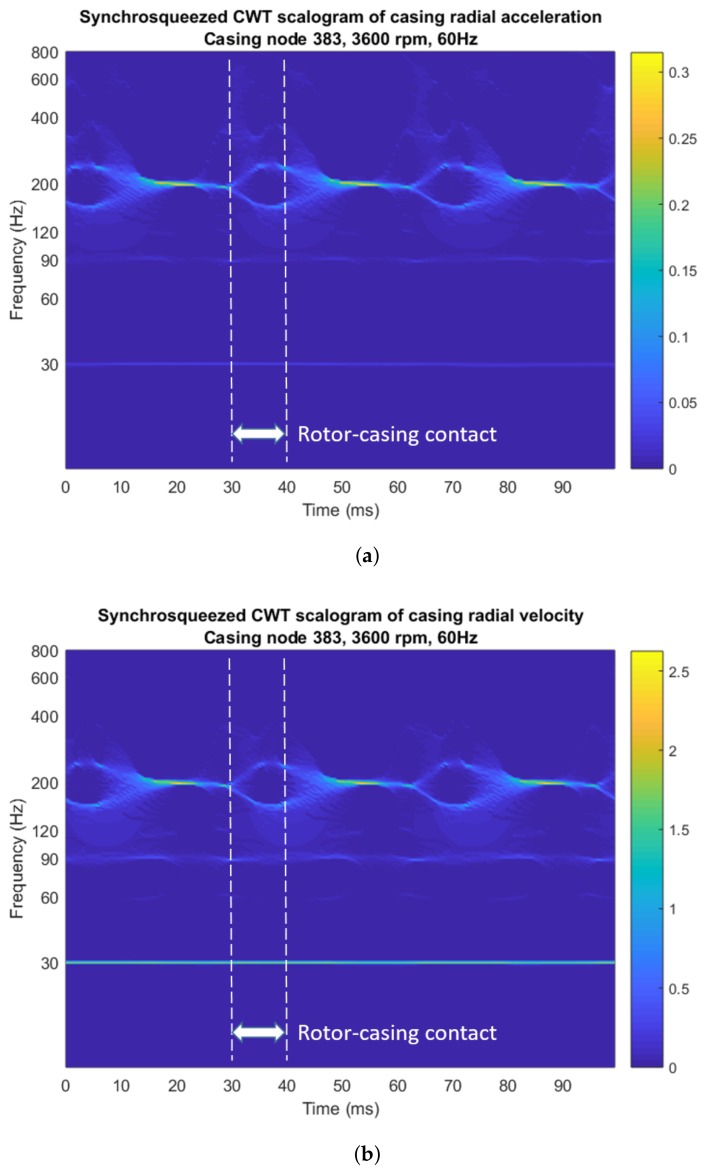
Synchrosqueezed CWT scalograms of the steady-state casing signals with the Morlet wavelet for single-point rub and the frequency axis in logarithmic scale: (**a**) casing acceleration, color scale in *g*; and (**b**) casing velocity, color scale in mm/s. The boundaries of one of the rotor–casing rubs are marked with white dotted lines.

The acceleration and velocity data were decomposed into wavelet coefficients with the Morlet wavelet function. First, the 2 s long time records were low-pass filtered and resampled to 3.2 kHz, resulting in two sampling vectors with 8000 samples. Then, only the last 160 samples of both vectors—with a length of 0.1 s, equivalent to six shaft rotation cycles at 3600 rpm—were picked for the calculation of the wavelet coefficients, discarding the rest. As mentioned above, a synchrosqueezing algorithm was applied to the coefficients for improved resolution. The 0–800 Hz acceleration and velocity scalograms representing coefficient absolute values with a colored scale are shown in Figure 12.

The two scalograms clearly highlight the fundamental rub frequency of 30 Hz and its lowest multiples, with the 3× harmonic—90 Hz, near the second shaft bending mode in Figure 6b—being the largest of them, and disclose the excitation of the 200 Hz casing vibration mode after the rub impact. The synchronous 60 Hz harmonic is very shady and therefore the scalograms hold no information about the rotor unbalance, which has been masked by the rub features. The localization of the rotor–casing contact event in time is possible because of these features: first, when rotor and rub obstacle touch, the 200 Hz band splits into two branches with different frequencies. This is a consequence of a change in the system modal characteristics when rotor and casing are mechanically coupled through their contact (See chapter 2 of [2]). Secondly, when rotor and casing contact ends, those two branches get slightly blurry before merging back into one 200 Hz band. Finally, the acceleration scalogram in Figure 12a shows two faint tongues in the 400–600 Hz band right at the boundaries of rotor–casing contact. These three signal features characterize rotor–casing rub, allow to localize the rub event in time and are not visible to the Fourier spectra in Figure 10. Edge effects are negligible.

#### Detection Times with DFT/FFT and CWT

Table 2 brings an estimation of the rub detection time with the Fourier spectrum and with CWT. The estimated time is the sum of the data acquisition time, equal to the time length of the vector of samples, plus the calculation time of the Fourier/wavelet coefficients during the data processing phase. These data processing times are the result of averaging the times of ten executions of the Matlab functions *fft* and *wsst* with the samples inputs.

Even though the CWT input vectors, with 160 samples, are far shorter than the DFT input vectors, with 56,000 (acceleration) and 4000 samples (velocity), the calculation of CWT coefficients is considerably slower than the calculation of the spectra. Nevertheless, since a more effective detection of rub can be carried out with much shorter sampling vectors, with lower acquisition times, the balance tips on the side of the CWT as a preferable monitorization tool.

## 4. Discussion

In this work, we presented a model of an aeroderivative gas turbine for the simulation of abnormal duty. In particular, it succeeds in reproducing a common type of rub encountered in conventional turbomachinery, the supercritical partial single-point rub with period-two (See chapter 2 of [2]). This is the first time that this rub has been studied in the context of aeroderivative gas turbines, with a model able to mimic either a compressor or turbine section. In addition, for the first time, the features of casing vibration have been extracted and analyzed as the available source of condition information and of diagnosis of this type of rub.

This rub is reflected as impulse trains in the casing acceleration and velocity signals (Figure 9). Each impulse marks the beginning or the end of rotor–casing contact. Since each one of those sharp peaks supposes a perturbation of the casing, they are followed by transient oscillations containing a number of rotor and casing vibration modes. the most notorious of those transients is the rigid solid casing mode in Figure 5c: the lowest of all the casing natural modes, and, therefore, the least affected by damping.

The Fourier spectra of the signals are mainly comprised of impulse trains with fundamental frequency equal to the impact frequency: half the rotor rotation speed (Figure 10). The highest impulse harmonics in the spectra are the fundamental frequency of 30 Hz and the peaks lying next to the 200 Hz casing natural mode. This vibration amplification near a casing natural frequency due to rotor–casing rub has been observed in [35]. The cepstrum analysis of these spectra yields new trains of impulses with fundamental frequency equal to the rub frequency (Figure 11), evidencing the periodicity of the rotor–casing rub.

The Fourier spectra and the cepstra in Figure 10 and Figure 11, due to their averaging nature and the small frequency resolution (0.5 Hz), fail to make visible the rotor and casing transient modes excited by the rub perturbation. This elements can instead be identified and precisely localized in time with the application of the Continuous Wavelet Transform on the signals (Figure 12). Because of the very large time resolution of the CWT at high frequencies, the analysis of a short vector of acceleration samples containing just a few of the rotor–casing impacts suffices to confirm the presence of those brief vibration transients and to localize them at the two ends of each rotor–casing contact, confirming the existence of rotor–casing rub. The CWT scalograms of the velocity data are better suited to visualize the low-frequency features of the rotor–casing rub, including the fundamental rub frequency band and some of its low-band multiples.

The visualization of those short-lived pieces of evidence of rotor–casing rub in a Fourier spectrum would require collecting very large sampling vectors with large time intervals between the first and the last sample to improve frequency resolution as much as needed without worsening the spectrum frequency range. The acquisition of large data sets is time-consuming and comes with a loss of precocity in the diagnosis. On the other hand, the Continuous Wavelet Transform has the potential to identify rotor–casing rub with the analysis of only a few impacts in a very short time span with no more than a small processing time penalty, as shown in Table 2. This enables early detection of this malfunction.

## Figures and Tables

**Table 1:** Rotor–casing model dimensions and mechanical properties.

Shaft length:	0.6 m	Young modulus of shaft:	$2\times 10^{11}$ Pa
Distance between shaft bearings:	0.4 m	Shaft density:	7850 kg m${}^{-3}$
Shaft diameter:	0.01 m	Casing density:	1600 kg m${}^{-3}$
Disk mass:	1.5 kg	Young modulus of casing:	7$\times 10^{10}$ Pa
Disk thickness:	0.025 m	Poisson’s ratio of casing:	0.1
Disk diameter:	0.1 m	Bearing radial direct stiffness:	1×$10^{8}$ N m${}^{-1}$
Casing midplane diameter:	0.134 m	Casing support stiffness:	5×$10^{5}$ N m${}^{-1}$
Casing length:	0.4 m	Bearing-casing truss bar stiffness:	1×$10^{12}$ N m${}^{-1}$
Casing thickness:	0.003 m	Bearing mass:	0.02 kg

**Table 2:** Comparison of time performances for rub fault detection.

		Time for Data Acquisition	Time for Data Processing	Total Time
	Acceleration data	2 s	0.001172 s	2.00117 s
DFT/FFT	Velocity data	2 s	0.000132 s	2.00013 s
	Acceleration data	0.1 s	0.026428 s	0.12643 s
CWT	Velocity data	0.1 s	0.009997 s	0.11000 s

## Abbreviations

*x*	Global radial horizontal axis
*y*	Global radial vertical axis
*z*	Global axial axis
M	Global mass matrix
C	Global viscous damping matrix
Ω	Rotor rotation speed
G	Global gyroscopic matrix
K	Global stiffness matrix
u	Vector of global nodal displacements
$u^{\prime }$	Vector of global nodal velocities
$u^{\prime \prime }$	Vector of global nodal accelerations
*t*	time variable
$F_{U}$	Global unbalance forces vector
$F_{R}$	Global rub forces vector
$M_{S+D}$	Shaft and disks mass submatrix
$M_{C}$	Casing mass submatrix
$M_{B}$	Bearing mass submatrix
$G_{S+D}$	Shaft and disks gyroscopic submatrix
$C_{S+D}$	Shaft and disks viscous damping submatrix
$C_{S+D,B}$, $C_{B,S+D}$	Viscous damping submatrices, shaft and disks-bearing coupling
$C_{C}$	Casing viscous damping submatrix
$C_{C,B}$, $C_{B,C}$	Viscous damping submatrices, casing-bearing coupling
$C_{B}$	Casing viscous damping submatrix
$K_{S+D}$	Shaft and disks viscous damping submatrix
$K_{S+D,B}$, $K_{B,S+D}$	Stiffness submatrices, shaft and disks-bearing coupling
$K_{C}$	Casing viscous damping submatrix
$K_{C,B}$, $K_{B,C}$	Stiffness submatrices, casing-bearing coupling
$K_{B}$	Bearing viscous damping submatrix
$u_{S+D}$	Vector of global shaft and disks nodal displacements
$u_{C}$	Vector of global casing nodal displacements
$u_{B}$	Vector of global bearing nodal displacements
$C_{M}$	Mass matrix coefficient of the Rayleigh damping equation
$C_{K}$	Stiffness matrix coefficient of the Rayleigh damping equation
$\xi_{d,i}$	damping factor of *i*-th system natural mode
$\omega_{N,i}$	*i*-th system natural frequency
$F_{U,i}$	Unbalance force vector at shaft node *i*
$M_{U,i}$	Unbalance mass at shaft node *i*
$r_{U,i}$	Unbalance mass distance to rotating axis at shaft node *i*
$\psi_{U,i}$	Angular position of the unbalance mass at shaft node *i*
$k_{R}$	Rub contact stiffness coefficient
$c_{R}$	Rub contact damping coefficient
$F_{R}$	Normal rub force
μ	Rub friction coefficient
$v_{2,j}$	Unitary vector normal to shell midplane at casing node j
$d_{R}$	Relative displacement between rotor and rub obstacle
$d_{R}^{\prime }$	Relative velocity between rotor and rub obstacle
$u_{x,i}$, $u_{y,i}$	Radial displacements of shaft node *i*
$u_{x,j}$, $u_{y,j}$	Radial displacements of casing node *j*
$u_{x,i}^{\prime }$, $u_{y,i}^{\prime }$	Radial velocities of shaft node *i*
$u_{x,j}^{\prime }$, $u_{y,j}^{\prime }$	Radial velocities of casing node *j*
ϵ	Clearance between rotor and rub obstacle
$R_{c}$	Casing midplane radius
$R_{r,i}$	Rotor radius at shaft node *i*
$F_{R,i}$, $F_{R,j}$	Rub force vectors at shaft and casing nodes *i* and *j*
γ	Angle of the unitary vector normal to shell midplane with respect to *x*
x	Vector of samples
X	Vector of Fourier coefficients
x(t)	Signal in the time domain
F	Fourier transform operator
$F^{-1}$	Inverse Fourier transform operator
c(t)	Cepstrum of a signal
Ψ	Mother wavelet function
*a*	Dilation factor of the Wavelet transform
*b*	Translation factor of the Wavelet transform
W(a,b)	Wavelet coefficient
*E*	Young modulus
*I*	Second moment of area
$L_{s}$	Shaft length between bearings
$u_{B}$	Boundary degrees of freedom
$u_{I}$	Internal degrees of freedom
$F_{B}$	Vector of external forces at the boundary degrees of freedom
$F_{I}$	Vector of external forces at the internal degrees of freedom
$M_{\mathrm{BB}}$, $M_{\mathrm{BI}}$, $M_{\mathrm{IB}}$, $M_{\mathrm{II}}$	Submatrices of the reordered mass matrix
$G_{\mathrm{BB}}$, $G_{\mathrm{BI}}$, $G_{\mathrm{IB}}$, $G_{\mathrm{II}}$	Submatrices of the reordered gyroscopic matrix
$C_{\mathrm{BB}}$, $C_{\mathrm{BI}}$, $C_{\mathrm{IB}}$, $C_{\mathrm{II}}$	Submatrices of the reordered viscous damping matrix
$K_{\mathrm{BB}}$, $K_{\mathrm{BI}}$, $K_{\mathrm{IB}}$, $K_{\mathrm{II}}$	Submatrices of the reordered stiffness matrix
$\Phi_{\mathrm{CB}}$	Craig–Bampton reduction matrix
$\phi_{I}$	Vector of modal internal degrees of freedom
$\Phi_{C}$	Submatrix of constrained modes
$\Phi_{N}$	Submatrix of matrix modes
Λ	Diagonal matrix of eigenvalues
$M_{\mathrm{CB}}$	Reduced mass matrix
$G_{\mathrm{CB}}$	Reduced gyroscopic matrix
$C_{\mathrm{CB}}$	Reduced viscous damping matrix
$K_{\mathrm{CB}}$	Reduced stiffness matrix
$F_{U,\mathrm{CB}}$	Reduced unbalance forces vector
$F_{R,\mathrm{CB}}$	Reduced rub forces vector
$F_{\mathrm{CB},R}$	Reduced global rub forces vector

## Appendix A. Description of the Craig–Bampton Method

The aim of this method is to reduce the number of degrees of freedom of the rotor–flexible casing system, represented by Equation (1).

The first step is to split the system degrees of freedom, contained in the modal displacements vector u, into two categories: the *boundary* degrees of freedom (subindex *B*) and the *interior* degrees of freedom (subindex *I*). The boundary degrees of freedom are those on which either boundary conditions or external forces are applied, and the interior degrees of freedom are the rest. This division prompts a reordering of the nodal displacements vector and its derivatives, the external force vectors and the system matrices as follows:

$$\begin{array}{c} \begin{array}{cc} u={\left(u_{B}|u_{I}\right)}^{T} & F={\left(F_{B}|F_{I}\right)}^{T}\to {\left(F_{B}|0\right)}^{T}\\ M=\left(\begin{array}{c} M_{\mathrm{BB}}\;M_{\mathrm{BI}}\\ M_{\mathrm{IB}}\;M_{\mathrm{II}} \end{array}\right) & G=\left(\begin{array}{c} G_{\mathrm{BB}}\;G_{\mathrm{BI}}\\ G_{\mathrm{IB}}\;G_{\mathrm{II}} \end{array}\right)\\ C=\left(\begin{array}{c} C_{\mathrm{BB}}\;C_{\mathrm{BI}}\\ C_{\mathrm{IB}}\;C_{\mathrm{II}} \end{array}\right) & K=\left(\begin{array}{c} K_{\mathrm{BB}}\;K_{\mathrm{BI}}\\ K_{\mathrm{IB}}\;K_{\mathrm{II}} \end{array}\right). \end{array} \end{array}$$

Note that the vector of external forces at the interior degrees of freedom is null, because by definition there are no external forces applied on them.

The core of the Craig–Bampton method is the orthogonal projection of the state space of the original system, represented by the reordered nodal displacements vector and its derivatives, over a reduced subspace with fewer degrees of freedom. Naming the Craig–Bampton reduction matrix $\Phi_{\mathrm{CB}}$, the projection of the nodal displacements vector is:

$$\begin{array}{c} u_{\mathrm{CB}}=\Phi_{\mathrm{CB}}^{T}\cdot u={\left(u_{B}|\phi_{I}\right)}^{T}. \end{array}$$

The projection retains the boundary degrees of freedom in $u_{B}$ and yields a set of *modal* degrees of freedom: $\phi_{I}$. The Craig–Bampton reduction matrix is:

$$\begin{array}{c} \Phi_{\mathrm{CB}}=\left[\begin{array}{cc} I & 0\\ \Phi_{C} & \Phi_{N} \end{array}\right]. \end{array}$$

I is an identity submatrix. $\Phi_{C}$ is the submatrix of *constrained modes*, calculated as:

$$\begin{array}{c} \Phi_{C}=-K_{\mathrm{II}}^{-1}\cdot K_{\mathrm{IB}}. \end{array}$$

Each *i*-th column of $\Phi_{C}$ represents the displacement of the interior degrees of freedom when a unitary displacement is set at the *i*-th boundary degree of freedom while the remaining boundary displacements are set to zero.

The final component of $\Phi_{\mathrm{CB}}$ is the submatrix of *matrix modes*, $\Phi_{N}$. Each of its columns is a modal shape of the system with the displacements of the boundary degrees of freedom constrained to zero. Those modal shapes are solutions of an eigenproblem formed by the interior stiffness and mass submatrices:

$$\begin{array}{c} K_{\mathrm{II}}\cdot \Phi_{N}=M_{\mathrm{II}}\cdot \Phi_{N}\cdot \Lambda , \end{array}$$

where Λ is the diagonal matrix with the eigenvalues. It is usual to normalize the columns of $\Phi_{N}$ so that the generalized modal mass equals one for every modal shape. This condition is expressed in matric notation as:

$$\begin{array}{c} \Phi_{N}^{T}\cdot M_{\mathrm{II}}\cdot \Phi_{N}=I. \end{array}$$

The system reduction is attained when only a few of the system matrix modes are retained in the submatrix $\Phi_{N}$.

The system global matrices are now projected over the Craig–Bampton subspace:

$$\begin{array}{c} \begin{array}{c} M_{\mathrm{CB}}=\Phi_{\mathrm{CB}}^{T}\cdot M\cdot \Phi_{\mathrm{CB}}\\ G_{\mathrm{CB}}=\Phi_{\mathrm{CB}}^{T}\cdot G\cdot \Phi_{\mathrm{CB}}\\ C_{\mathrm{CB}}=\Phi_{\mathrm{CB}}^{T}\cdot C\cdot \Phi_{\mathrm{CB}}\\ K_{\mathrm{CB}}=\Phi_{\mathrm{CB}}^{T}\cdot K\cdot \Phi_{\mathrm{CB}}. \end{array} \end{array}$$

The projection of the external force vectors results in vectors that conserve their values at the boundary degrees of freedom, while the forces at the interior degrees of freedom remain zero. Their product by $\Phi_{\mathrm{CB}}^{T}$ simply shortens them as the projection subspace is smaller than the original state space:

$$\begin{array}{c} \begin{array}{c} F_{U,\mathrm{CB}}=\Phi_{\mathrm{CB}}^{T}\cdot F_{U}={\left[F_{U,1}\ldots F_{U,N}\right]}^{T}\\ F_{R,\mathrm{CB}}=\Phi_{\mathrm{CB}}^{T}\cdot F_{R}={\left[F_{R,1}\ldots F_{R,N}\right]}^{T}, \end{array} \end{array}$$

with *N* representing here the number of degrees of freedom, or dimension, of the Craig–Bampton subspace.

The reduced rotor–casing system of differential equations can now be assembled:

$$\begin{array}{c} M_{\mathrm{CB}}u_{\mathrm{CB}}^{\prime \prime }+\left(C_{\mathrm{CB}}+\Omega G_{\mathrm{CB}}\right)u_{\mathrm{CB}}^{\prime }+K_{\mathrm{CB}}u_{\mathrm{CB}}=F_{U,\mathrm{CB}}\left(t\right)+F_{R,\mathrm{CB}}\left(u,u^{\prime }\right). \end{array}$$
